# Data on monogenean (Platyhelminth) parasites in 11 populations of *Astyanax aeneus* (Pisces: Teleostei) in a neotropical river in Chiapas, south Mexico

**DOI:** 10.1016/j.dib.2019.103936

**Published:** 2019-04-24

**Authors:** Guillermo Salgado-Maldonado, Edgar F. Mendoza-Franco, Juan Manuel Caspeta-Mandujano, Carlos Ramírez-Martínez

**Affiliations:** a Universidad Nacional Autónoma de México, Instituto de Biología, Laboratorio de Helmintología, Apartado Postal 70-153, CP 04510, Ciudad de México, Mexico; b Universidad Autónoma de Campeche, Instituto de Ecología, Pesquerías y Oceanografía del Golfo de México (EPOMEX), San Francisco de Campeche, Campeche, Mexico; c Universidad Autónoma del Estado de Morelos, Facultad de Ciencias Biológicas, Laboratorio de Parasitología de Animales Silvestres, Cuernavaca, Morelos, Mexico; d Universidad Autónoma de Nuevo León, Facultad de Medicina Veterinaria y Zootecnia, Monterrey, Nuevo León, Mexico

## Abstract

The data presented in this article are related to the research article entitled “Aggregation and negative interactions in low-diversity and unsaturated monogenean (Platyhelminthes) communities in *Astyanax aeneus* (Teleostei) populations in a neotropical river of Mexico” publicated in Int. J. Parasitol. Parasites Wildl. 8 (2019) 203–215. https://doi.org/10.1016/j.ijppaw.2019.02.005. This article describes the communities of monogenean parasites in 11 populations of a small characid freshwater fish *Astyanax aeneus* (Günther) separated by small geographical distances along 60 km of the Lacantún river in Chiapas, Mexico. We examined 15 *A. aeneus* from each of 11 locations (one sample in February, a second sample in August 2012), situated at the mouth of the streams opening into the main body of the Lacantún river, at the Montes Azules Biosphere Reserve in the Lacandon forest, Chiapas in southern Mexico. The area of study is located ∼800 km from the mouth of the Usumacinta river in the Gulf of Mexico. In this paper we provide the data for 12 monogenean taxa. The material in this Data in Brief paper comprised the raw data on the abundance distribution of each monogenean taxa recorded in each of the locations; i. e. the number of helminth individuals of each of 12 taxa found in each one individual of *A. aeneus* from each of 11 localities. The data set is contained in a single table text document including one matrix per date of collection and locality of monogenean species (lines) per host *A. aeneus* (columns).

**Table:** **Specifications table***[please fill in right-hand column of the table below]*

Subject area	*Biology*
More specific subject area	*Platyhelminth parasites, monogeneans of tropical freshwater fish*
Type of data	*Table (text document)*
How data was acquired	*Microscope, survey. Each fish was examined under a stereo microscope in Petri dishes with river water. Skin, scales, mouth, branchial cavity, anus, and fins of each host were examined. Fish were euthanized and the branchial arches were removed, separated from the brachial cavity and evaluated individually. We collected data on the number of species (species richness) and abundance distribution of monogeneans (num. of individuals of each species).*
Data format	*Raw numbers in text document; matrices of monogenean taxa (lines) recorded in each one fish host individual (columns); one matrix per each one of 11 locations*
Experimental factors	*We examined 158 fish Astyanax aeneus during February and 150 during August 2012; in total we examined 308 specimens of A. aeneus.* *Fish were collected using gill nets, transferred to the laboratory and kept alive in aerated containers until they were examined for monogeneans, performed within 8 hours of capture. Each fish was measured (total and standard length; maximum deep) and examined under a stereo microscope in Petri dishes with river water. Skin, scales, mouth, branchial cavity, anus, and fins of each host were examined. Fish were euthanized and the branchial arches were removed, separated from the brachial cavity and evaluated individually.*
Experimental features	*All monogeneans found were fixed in 4% hot formaldehyde, stained with Gomorís triple stain and mounted on Canada balsam. Taxonomic identification was performed based on morphometric analysis of the specimens.*
Data source location	*MEXICO (south of the country), state of Chiapas. Eleven sample locations situated on the opening of streams tributaries to the main Rio Lacantún in the Biosphere Reserve Montes Azules (RBMA), Chiapas, México:*[1]*Río Tzendales (16°17′ 10.8″ N; 90°53′12.6″ W),*[2]*Río Manzanares (16°10′14.6″ N; 90°50′36.2″ W),*[3]*Arroyo Miranda (16°08′08.1″ N; 90°55′14.9″ W),*[4]*Río Danta (16°09′08.1″ N; 90°54′06.3″ W), (5) Arroyo Lagarto (16°08′14.0″ N; 90°54′24.4″ W), (6) Embarcadero Estación Chajul (16°06′38.4″ N; 90°56′ 23.6″ W), (7) Arroyo José (16°06′50″ N; 90°56′03.3″ W), (8) Río Chajul (16°05′58.2″ N; 90°57′30.1″ W), (9) Río San Pablo (16°06′ 10.0″ N; 91°00′52.2″ W), (10) Río Puerto Rico (16°05′04.4″ N; 91°01′11.2″ W), (11) Río Ixcan (16°07′17.5″ N; 91°05′11.3″ W).*
Data accessibility	*Data provided within this article.*
Related research article	*Salgado-Maldonado, G., Mendoza-Franco, E. F., Caspeta-Mandujano, J. M., Ramírez-Martínez, C. Aggregation and negative interactions in low-diversity and unsaturated monogenean (Platyhelminthes) communities in Astyanax aeneus (Teleostei) populations in a neotropical river of Mexico. Int.l J Parasitol. Parasites Wildl.* 8 (2019) 203–215. https://doi.org/10.1016/j.ijppaw.2019.02.005

**Table:** 

**Value of the data** • These data will assist to examine spatial variation in community structure of helminth parasites of freshwater fishes. • These data could support to explore characteristics of the structure of parasite assemblages as nestedness or patterns of decay of similarity with distance. • The data set may also assist to compare patterns of structure of assemblage vs appropriate null models. • The data set can be useful to compare population or community characteristics i. e. richness, densities, of tropical assemblages vs temperate or other regions.

## 1 Data

This raw data incorporates a comprehensive survey of 12 taxa of monogenean (Platyhelminthes) parasites in 308 individuals of 11 populations of a freshwater tropical fish Astyanax aeneus distributed along 60 km of a tropical river in south Mexico; we examined 158 fish during February and 150 during August 2012.

### 2 Experimental design, materials and methods

We examined 15 *A. aeneus* from each of 11 locations, situated at the mouth of the streams opening into the main body of the Lacantún river, at the Montes Azules Biosphere Reserve in the Lacandon forest, Chiapas in southern Mexico. This river belongs to the Usumacinta river watershed. The area of study is located ∼800 km from the mouth of the Usumacinta river in the Gulf of Mexico (see [3]). We examined 158 fish during February and 150 during August 2012; in total we examined 308 specimens of *A. aeneus*. Eleven sample locations situated on the opening of streams tributaries to the main Rio Lacantún in the Biosphere Reserve Montes Azules (RBMA), Chiapas, México were chosen as follows [1]: Río Tzendales (16°17′ 10.8″ N; 90°53′12.6″ W) [2], Río Manzanares (16°10′14.6″ N; 90°50′36.2″ W) [3], Arroyo Miranda (16°08′08.1″ N; 90°55′14.9″ W) [4], Río Danta (16°09′08.1″ N; 90°54′06.3″ W), (5) Arroyo Lagarto (16°08′14.0″ N; 90°54′24.4″ W), (6) Embarcadero Estación Chajul (16°06′38.4″ N; 90°56′ 23.6″ W), (7) Arroyo José (16°06′50″ N; 90°56′03.3″ W), (8) Río Chajul (16°05′58.2″ N; 90°57′30.1″ W), (9) Río San Pablo (16°06′ 10.0″ N; 91°00′52.2″ W), (10) Río Puerto Rico (16°05′04.4″ N; 91°01′11.2″ W), (11) Río Ixcan (16°07′17.5″ N; 91°05′11.3″ W).

Fish were collected using gill nets, transferred to the laboratory and kept alive in aerated containers until they were examined for monogeneans, performed within 8 hours of capture. Each fish was measured (standard length) and examined under a stereo microscope in Petri dishes with river water. Skin, scales, mouth, branchial cavity, anus, and fins of each host were examined. Fish were euthanized and the branchial arches were removed, separated from the brachial cavity and evaluated individually (protocol for the use of fish in research based on the NORM – 019 – STPS – 1993 established by the Instituto de Ecología, Pesquerías y Oceanografía del Golfo de México EPOMEX, Campeche, Mexico; specimens collected under the Cartilla Nacional de Colector Científico FAUT-0105 issued by the Secretaría del Medio Ambiente y Recursos Naturales [SEMARNAT] to GSM). All monogeneans found were fixed in 4% hot formaldehyde, stained with Gomori's triple stain and mounted on Canada balsam.

Taxonomic identification was performed based on morphometric analysis of the specimens. A list of monogenean taxa, habitat (organ of the fish where it was found) and numbers of museum catalog of deposited voucher specimens is presented in Table 1 (see [1, 2]).

**Table 1:** Monogenean parasites of *Astyanax aeneus* from Río Lacantún, Chiapas, Mexico. Voucher specimens were deposited in the Colección Nacional de Helmintos (CNHE), Universidad Nacional Autónoma de México, Instituto de Biología.

	Habitat	CNHE Catalog No.
Dactylogyridae		
*Cacatuocotyle chajuli* Mendoza-Franco, Caspeta-Mandujano and Salgado-Maldonado, 2013	External surface of anal opening	8268; 8269; 8270; 8276
*Cacatuocotyle exiguum* Mendoza-Franco, Caspeta-Mandujano and Salgado-Maldonado, 2013	Gill lamellae	8277; 8278; 8279
*Cacatuocotyle* sp.	Gill lamellae	8280
*Characithecium costaricensis* (Price and Bussing, 1967)	Gill lamellae	6274–6276
*Diaphorocleidus kabatai* (Molnar, Hanek and Fernando, 1974)	Gill lamellae	6281
*Palombitrema heteroancistrium* (Price and Bussing, 1968)	Gill lamellae	6279–6280
*Urocleidoides strombicirrus* (Price and Bussing, 1967)	Gill lamellae	
Dactylogyridae gen. sp.	Gill lamellae	
Gyrodactylidae		
*Anacanthocotyle anacanthocotyle* Kritsky and Fritts, 1970	Gill lamellae	
*Anacanthocotyle* sp.,	Gill lamellae	
*Gyrodactylus neotropicalis* Kritsky and Fritts, 1970	Fins	
*Gyrodactylus* sp.	Fins	

In Table 2 (text document) we show for each locality the number of monogenean individuals of each taxa (lines) recorded in each fish individual examined (columns), referred to each locality and date of collection.

**Table 2:** Raw data on Monogenean parasites of *Astyanax aeneus* from Río Lacantún, Chiapas, Mexico. Each sampled locality identified by name and coordinates, was sampled during February and August 2012; measures (total length, standard length, maximum deep, sex of specimens was documented for some collections) for each one of 15 fish examined each sampled period from each locality are given. Data include the number of individual monogeneans of each taxa collected by each examined fish.

**Locality: Río Tzendales 16°17′ 10.8″ N; 90°53′12.6″ W**																
date of collection: February 23, 2012																
*Astyanax aeneus* #. →		1	2	3	4	5	6	7	8	9	10	11	12	13	14	15
Total length		47	50	53	57	56	65	65	54	50	46	40	45	53	51	48
Standard length		39			50	47	55		44	40		35		42	42	
Maximum deep		10	11	15	15	12	15	18	12	10	13	9	11	12	13	13

**Monogeneans:**																
*A. anacanthocotyle*																1
*C. chajuli*			1													1
*D. kabatai*																1
*P. heterancisthrium*		1							1		1					

**Locality: Río Tzendales 16°17′ 10.8″ N; 90°53′12.6″ W**																
date of collection: August 8, 2012																
*Astyanax aeneus* #. **→**		1	2	3	4	5	6	7	8	9	10	11	12	13	14	15
Total length		84	55	56	79	60	88	55	56	85	60	70	55	35	55	50
Standard length		68	45	45	63	50	70	46	46	72	49	56	45	30	44	44
Maximum deep		25	10	9	23	9	23	9	9	23	15	18	14	7	14	14
Sex			♂	♀		♀		♀	♀	♀	♀		♂			♀

**Monogeneans:**																
*A. anacanthocotyle*		1	2	2	1			2	3		2		2	1		1
*C. chajuli*							1									
*C. exiguum*							2									
*Ch. costariscensis*		2	2	1	1		27	1		2		2			3	
*D. kabatai*		1					1		1		1	2				
*P. heterancisthrium*		3			1	1	9	1		1					6	

**Locality: Río Manzanares 16°10′14.6″ N; 90°50′36.2″ W**																
date of collection: February 22, 2012																
*Astyanax aeneus* #. **→**		1	2	3	4	5	6	7	8	9	10	11	12	13	14	15
Total length		42	44	90	41	41	45	41	56	39	38	44	39	100	38	39
Standard length		35		75	36	35		36		34	34		35	87	34	35
Maximum deep		10	10	24	9	9	11	10	14	9	9	10	10	30	10	9

**Monogeneans:**																
*A. anacanthocotyle*		4	3				4			3		1		2		
*C. chajuli*										1	1	3	3		1	
*C. exiguum*							1					1				
*Ch. costariscensis*					2		4	4	13			3	3		4	
*D. kabatai*												1				

**Locality: Río Manzanares 16°10′14.6″ N; 90°50′36.2″ W**																
date of collection: August 7, 2012																
*Astyanax aeneus* #. **→**		1	2	3	4	5	6	7	8	9	10	11	12	13	14	15
Total length		53	73	60	102	31	35	35	77	54	57	53	65	97	68	48
Standard length		43	54	45	85	28	27	28	63	45	47	47	55	80	54	30
Maximum deep		18	15	17	30	8	9	7	23	13	12	10	17	30	18	16
Sex			♂		♀	juv	juv	juv		♀	♀	♀	♂	♀		♀

**Monogeneans:**																
*A. anacanthocotyle*		1		1	1	2		6			1	4	1	1	2	2
*C. exiguum*															1	
*Ch. costariscensis*		3		1			2		17	2			2	7	10	
*P. heterancisthrium*			1													

**Locality: Arroyo Miranda 16°08′08.1″ N; 90°55′14.9″ W**																
date of collection: February 21, 2012																
*Astyanax aeneus* #. **→**		1	2	3	4	5	6	7	8	9	10	11	12	13	14	15
Total length		42	55	60	42	38	35	40	35	42	38	40	37	36	67	44
Standard length		35	45	49	36		28	35	30		34	34		31	57	38
Maximum deep		10	14	15	11	12	9	10	8	12	10	11	10	10	18	10

**Monogeneans:**																
*Ch. costariscensis*		2			2	2	1	1		1						
*D. kabatai*						1										
*P. heterancisthrium*								1								

**Locality: Arroyo Miranda 16°08′08.1″ N; 90°55′14.9″ W**																
date of collection: August 7, 2012																
*Astyanax aeneus* #. **→**		1	2	3	4	5	6	7	8	9	10	11	12	13	14	15
Total length		60	90	100	102	29	100	60	50	58	30	30	32	30	29	48
Standard length		50	76	80	85	24	85	51	42	48	25	23	25	24	23	30
Maximum deep		15	30	33	30	5	30	15	10	17	5	7	7	6	6	16
Sex		♂	♀	♀			♀	♂	♀	juv	juv	juv	juv	juv	juv	juv

**Monogeneans:**																
*A. anacanthocotyle*					5						6	4	2	2		
*Ch. costariscensis*					7		1		1	4		3	1	3		6
*P. heterancisthrium*			2	4	4		1		1	4						1

**Locality: Rio Danta 16°09′08.1″ N; 90°54′06.3″ W**																
date of collection: February 22, 2012																
*Astyanax aeneus* #. **→**	1	2	3	4	5	6	7	8	9	10	11	12	13	14	15	
Total length	80	60	41	59	65	80	48	66	75	55	66	56	71	68	67	
Standard length		50	36		53		42		60		47		60	48		
Maximum deep	24	15	12	15	20	20	9	18	21	13	18	15	22	19	16	

**Monogeneans:**																
*A. anacanthocotyle*		5						1								
*C. chajuli*												3			1	
*Ch. costariscensis*	5		2			6	1	1	1	4	2	2	2			

**Locality: Rio Danta 16°09′08.1″ N; 90°54′06.3″ W**																
date of collection: August 7, 2012																
*Astyanax aeneus* #. **→**	1	2	3	4	5	6	7	8	9	10	11	12	13	14	15	
Total length	105	97	100	75	110	90	81	110	90	112	53	85	86	85	48	
Standard length	90	80	87	63	94	76	70	90	80	95	47	68	68	73	30	
Maximum deep	35	30	35	22	35	28	24	40	24	42	10	25	25	26	16	
Sex	♀	♂	♀		♀	♂		♀	♀	♀	♂					

**Monogeneans:**																
*A. anacanthocotyle*				1												
*C. exiguum*														1		
*Ch. costariscensis*	1	4	1	1	2	2	2		3		2	8		13	5	
*D. kabatai*							3					1		5		
*P. heterancisthrium*		1		3								6		7	8	

**Locality: Arroyo Lagarto 16°08′14.0″ N; 90°54′24.4″ W**																
date of collection: February 21, 2012																
*Astyanax aeneus* #. **→**	1	2	3	4	5	6	7	8	9	10	11	12	13	14	15	
Total length	43	42	42	42	42	43	42	40	43		45	43	38	37	38	
Standard length		33	30			35			37					30		
Maximum deep	11	9	9	9	10	10	9	8	9		11	12	9	10	10	

**Monogeneans:**																
*A. anacanthocotyle*			1			1										
*Cacatuocotyle chajuli*	2						1									
*Ch. costariscensis*	2			2	1			1	2							

**Locality: Arroyo Lagarto 16°08′14.0″ N; 90°54′24.4″ W**																
date of collection: August 6, 2012																
*Astyanax aeneus* #. **→**	1	2	3	4	5	6	7	8	9	10	11	12	13	14	15	
Total length	72	70	70	82	70	57	66	67	80	65	75	57	63	43	42	
Standard length	58	53	60	66	60	47	54	54	66	54	60	50	50	38	48	
Maximum deep	20	18	20	25	20	15	18	19	23	18	20	16	15	11	10	
Sex			♀		♀	♀		♂	♀		♀			♀	♀	

**Monogeneans:**																
*A. anacanthocotyle*				1	3				3	2			1	2		
*Ch. costariscensis*	3	1		1				2							1	
*D. kabatai*				1												
*P. heterancisthrium*	1		3							2	1					
Dactylogyridae gen. sp.														1		

**Locality: Embarcadero Estación Chajul 16°06′38.4″ N; 90°56′ 23.6″ W**																
date of collection: February 23, 2012																
*Astyanax aeneus* #. **→**	1	2	3	4	5	6	7	8	9	10	11	12	13	14	15	
Total length	42	38	46	47	39	40	44	36	45	35	55	37	40		40	
Standard length		32	37	40		30	39		39	30		34	32			
Maximum deep	11	9	10	10	10	10	9	9	9	8	8	8	10		9	

**Monogeneans:**																
*A. anacanthocotyle*				1				1				2				
*C. chajuli*		2														
*Ch. costariscensis*	1				1	2			1	1	1		1	2	1	
*D. kabatai*								1					1			
*P. heterancisthrium*						1										
**Locality: arroyo José 16°06′50″ N; 90°56′03.3″ W**																
date of collection: February 22, 2012																
*Astyanax aeneus* #. **→**	1	2	3	4	5	6	7	8	9	10	11	12	13	14	15	
Total length	50	55	44	38	74	35	62	45	47	47	45	43	37	52	50	
Standard length		47		30	60	30	52		39	40		38	32		40	
Maximum deep	13	12	11	10	20	9	12	12	11	12	12	10	9	13	13	

**Monogeneans:**																
*C. chajuli*	4					2										
*Ch. costariscensis*		2	2						2	2				1		
*D. kabatai*					1											

**Locality: arroyo José 16°06′50″ N; 90°56′03.3″ W**																
date of collection: August 9, 2012																
*Astyanax aeneus* #. **→**	1	2	3	4	5	6	7	8								
Total length	68	55	60	58	60	57	56	56								
Standard length	55	46	48	45	48	48	46	45								
Maximum deep	18	15	15	15	15	15	14	14								

**Monogeneans:**																
*A. anacanthocotyle*			1	2												
*C. chajuli*				1												
*Ch. costariscensis*				3				1								

**Locality: Río Chajul 16°05′58.2″ N; 90°57′30.1″ W**																
date of collection: February 20, 2012																
*Astyanax aeneus* #. **→**	1	2	3	4	5	6	7	8	9	10	11	12	13	14	15	
Total length	35	45	34	36	40	45	43	42	35	46	42	38	39	39	43	
Standard length	30		29	31	30	36	34		29		30	32	33		33	
Maximum deep	8	13	7	7	10	9	8	12	8	12	9	8	8	9	10	

**Monogeneans:**																
*A. anacanthocotyle*										1						
*C. chajuli*		2		2								1				
*Cacatuocotyle* sp. (gills)											1					
*Ch. costariscensis*	1	6			3	1		2	1	1	3	1	4	3		
*D. kabatai*										1	1			1		
*P. heterancisthrium*		2													1	
*U. strombicirrus*															1	

**Locality: Río Chajul 16°05′58.2″ N; 90°57′30.1″ W**																
date of collection: August 6, 2012																
*Astyanax aeneus* #. **→**	1	2	3	4	5	6	7	8	9	10	11	12	13	14	15	
Total length	60	72	53	42	36	50	65	55	54	57	65	53	47	58	48	
Standard length	49	60	40	30	30	40	54	47	43	48	52	41	38	45	38	
Maximum deep	18	20	14	10	8	10	17	20	14	20	19	14	10	16	10	
Sex	♀	♀	♂	♀	♀	♀		♀		♀	♀		♂		♀	

**Monogeneans:**																
*P. heterancisthrium*								3		4			1	2	1	

*Ch. costariscensis*		3	1			1								1		

**Locality: Río San Pablo 16°06′ 10.0″ N; 91°00′52.2″ W**																
date of collection: February 21, 2012																
*Astyanax aeneus* #. **→**	1	2	3	4	5	6	7	8	9	10	11	12	13	14	15	
Total length	63	42	66	58	39	42	43	44	46	48	43	65	41	42	75	
Standard length	56	36		47			37		39	38		56		38		
Maximum deep	14	9	17	12	10	12	9	12	11	11	10	15	11	10	19	

**Monogeneans:**																
*C. chajuli*		1														
*C. exiguum*		1														
*Ch. costariscensis*				2	3	2	1	4	1							

**Locality: Río San Pablo 16°06′ 10.0″ N; 91°00′52.2″ W**																
date of collection: August 7, 2012																
*Astyanax aeneus* #. **→**	1	2	3	4	5	6	7	8	9	10	11	12	13	14	15	
Total length	64	55	50	82	54	60	56	45	53	44	58	44	60	35	58	
Standard length	50	46	40	66	46	52	44	36	44	36	48	35	50	28	48	
Maximum deep	15	13	10	25	11	12	14	10	10	11	17	8	15	8	16	
Sex		♀	♀		♀	♀		♂	♀	♀		♀	♂	♀	♀	

**Monogeneans:**																
*A. anacanthocotyle*							1			1		1				
*Ch. costariscensis*	1	1		2			3		3	2	1			2	6	
*D. kabatai*							1									
*P. heterancisthrium*			2			1					1				1	

**Locality: Río Puerto Rico 16°05′04.4″ N; 91°01′11.2″ W**																
date of collection: February 19, 2012																
*Astyanax aeneus* #. **→**	1	2	3	4	5	6	7	8	9	10	11	12	13	14	15	
Total length	45	48	44	43	48	38	42	42	40	41	35	65	44	49	40	
Standard length			38	39		30	38		30	37	30	50		35	30	
Maximum deep	13	13	10	10	13	9	10	11	9	10	9	18	10	9	8	

**Monogeneans:**																
*A. anacanthocotyle*													1			
*G. neotropicalis*			1											3		
*C. chajuli*	2					1		1	1							
*C. exiguum*							1									
*Ch. costariscensis*	2	1			3			6		2	2	1	7			

**Locality: Río Puerto Rico 16°05′04.4″ N; 91°01′11.2″ W**																
date of collection: August 5, 2012																
*Astyanax aeneus* #. **→**	1	2	3	4	5	6	7	8	9	10	11	12	13	14	15	
Total length	60	58	62	55	56	57	60	53	50	64	65	57	63	43	42	
Standard length	49	49	50	45	47	46	48	42	43	58	50	50	50	38	48	
Maximum deep	13	16	14	18	16	17	14	10	9	14	15	16	13	11	10	
Sex	♀		♀			♀		♂	♂		♀			♀	♀	

**Monogeneans:**																
*A. anacanthocotyle*	1	1		2	2		1						1	2		
*Gyrodactylus* sp.	1															
*Ch. costariscensis*		4		4	4	1	3	2	5				5		1	
*D. kabatai*			1										1			
*P. heterancisthrium*			1	2			1			4	1		3			
Dactylogyridae gen. sp.														1		

**Locality: Río Ixcan 16°07′17.5″ N; 91°05′11.3″ W**																
date of collection: February 20, 2012																
*Astyanax aeneus* #. **→**	1	2	3	4	5	6	7	8	9	10	11	12	13	14	15	
Total length	42	40	45	43	42	47	45	42	39	46	52	45	49	39	42	
Standard length	34	31	35	33	31	41		33	34		43		33			
Maximum deep	10	9	13	10	10	12	13	11	9	12	12	12	10	10	12	

**Monogeneans:**																
*A. anacanthocotyle*							1	1			1					
*G. neotropicalis*											1					
*Ch. costariscensis*			14		2		5	1		4	2	3	4		5	
*D. kabatai*				1		2	1									
*P. heterancisthrium*			1				1									
*U. strombicirrus*			1						3							

**Locality: Río Ixcan 16°07′17.5″ N; 91°05′11.3″ W**																
date of collection: August 6, 2012																
*Astyanax aeneus* #. **→**	1	2	3	4	5	6	7	8	9	10	11	12	13	14	15	
Total length	56	55	66	62	63	50	60	50	43	67	47	52	50	43	42	
Standard length	49	44	53	48	54	40	48	41	36	60	38	42	40	38	48	
Maximum deep	20	14	22	16	14	12	14	12	9	15	10	15	11	11	10	
Sex	♀		♀		♂	♀		♂	♂	♀	♀			♀	♀	

**Monogeneans:**																
*A. anacanthocotyle*				2									1			
*Anacanthocotyle* sp.	1	1												1		
*Gyrodactylus* sp.	1															
*Ch. costariscensis*		3		4	4	2				3						
*D. kabatai*			1								11					
*P. heterancisthrium*	1		2	2							1	3				
Dactylogyridae gen. sp.														1	1	
