# Supplementary material for: Data on monogenean (Platyhelminth) parasites in 11 populations of Astyanax aeneus (Pisces: Teleostei) in a neotropical river in Chiapas, south Mexico
Source: Data Brief. 2019 Apr 24;24:103936. doi: 10.1016/j.dib.2019.103936 (PMC6535688; doi:10.1016/j.dib.2019.103936)
Supplement: Multimedia component 1 [file mmc1.docx]

March 21, 2019

**Conflict of interest declaration**

DIB-D-19-00507

**None**
